# Using the STROBE statement to assess reporting in blindness prevalence surveys in low and middle income countries

**DOI:** 10.1371/journal.pone.0176178

**Published:** 2017-05-08

**Authors:** Jacqueline Ramke, Anna Palagyi, Vanessa Jordan, Jennifer Petkovic, Clare E. Gilbert

**Affiliations:** 1 School of Population Health, Faculty of Medicine and Health Sciences, University of Auckland, Auckland, New Zealand; 2 The George Institute for Global Health, Sydney Medical School, University of Sydney, Sydney, New South Wales, Australia; 3 Department of Obstetrics and Gynaecology, Faculty of Medicine and Health Sciences, University of Auckland, Auckland, New Zealand; 4 Bruyère Research Institute, University of Ottawa, Ottawa, Canada; 5 International Centre for Eye Health, Clinical Research Unit, Department of Infectious & Tropical Diseases, London School of Hygiene and Tropical Medicine, London, United Kingdom; LV Prasad Eye Institute, INDIA

## Abstract

**Objective:**

Cross-sectional blindness prevalence surveys are essential to plan and monitor eye care services. Incomplete or inaccurate reporting can prevent effective translation of research findings. The *Strengthening the Reporting of Observational Studies in Epidemiology* (STROBE) statement is a 32 item checklist developed to improve reporting of observational studies. The aim of this study was to assess the completeness of reporting in blindness prevalence surveys in low and middle income countries (LMICs) using STROBE.

**Methods:**

MEDLINE, EMBASE and Web of Science databases were searched on April 8 2016 to identify cross-sectional blindness prevalence surveys undertaken in LMICs and published after STROBE was published in December 2007. The STROBE tool was applied to all included studies, and each STROBE item was categorized as ‘yes’ (met criteria), ‘no’ (did not meet criteria) or ‘not applicable’. The ‘Completeness of reporting (COR) score’ for each manuscript was calculated: COR score = yes / [yes + no]. In journals with included studies the instructions to authors and reviewers were checked for reference to STROBE.

**Results:**

The 89 included studies were undertaken in 32 countries and published in 37 journals. The mean COR score was 60.9% (95% confidence interval [CI] 58.1–63.7%; range 30.8–88.9%). The mean COR score did not differ between surveys published in journals with author instructions referring to STROBE (10/37 journals; 61.1%, 95%CI 56.4–65.8%) or in journals where STROBE was not mentioned (60.9%, 95%CI 57.4–64.3%; p = 0.93).

**Conclusion:**

While reporting in blindness prevalence surveys is strong in some areas, others need improvement. We recommend that more journals adopt the STROBE checklist and ensure it is used by authors and reviewers.

## Introduction

Cross-sectional prevalence surveys are essential to plan and monitor local and national eye care service delivery, as well as to monitor the prevalence and causes of blindness and visual impairment globally. The latest estimate of global blindness drew on 227 prevalence surveys from 84 countries undertaken between 1990 and 2012.[1] In the current global eye health action plan the World Health Organization (WHO) called for more prevalence surveys to be undertaken in low and middle income countries (LMICs) to enable evidence-informed planning.[2]

Resources for health research are scarce—especially in LMICs—so it is essential that research findings are translated into health care policy and practice.[3] Incomplete or inaccurate reporting of study methods and results hinders understanding of a study’s rigour, and limits interpretation and effective translation of the findings. The manuscripts reporting blindness and visual impairment prevalence surveys (hereafter referred to as blindness prevalence surveys) do not always allow the reader to understand “what was planned, what was done, what was found, and what conclusions were drawn”.[4] Incomplete reporting can also make synthesis of evidence difficult or impossible.[5]

To improve reporting and facilitate critical appraisal of observational studies, including cross-sectional surveys, the *Strengthening the Reporting of Observational Studies in Epidemiology* (STROBE) statement was developed from a collaboration of epidemiologists, methodologists, statisticians, researchers and journal editors.[4] The STROBE statement was disseminated in 2007 and provides a checklist of 32 items recommended for inclusion in the reporting of observational studies. The items include aspects of study design, sample selection, data collection, analysis, and potential bias.[4] To date it has been endorsed by more than 120 journals, including The Lancet and PLoS (see list at http://www.strobe-statement.org/).

This study aimed to assess the extent to which items in the STROBE cross-sectional checklist (available for download at http://www.strobe-statement.org/) were reported in blindness prevalence surveys published after STROBE was disseminated.

## Methods

The search and study selection process is outlined in Box 1.

**Box 1. Study search and selection process**

**Search**

Date—April 8 2016

Databases—MEDLINE, EMBASE and Web of Science

Algorithm—‘blindness or vis* impairment or low vision’ and *‘prevalence or rapid assessment or population-based’*.

Limits—Published between 1 January 2008 and 31 March 2016

Additional searching—Examined reference lists of published reviews of blindness prevalence[6–9]

Process—Two researchers screened the titles of all citations identified during the initial search and the full-text manuscript was retrieved for review if the citation was potentially relevant

**Selection criteria**

**Inclusion**
- conducted in countries classified by The World Bank as LMIC in 2014 (Gross National Income <US$8,498) [10]
- presented cross-sectional population-based data
- provided information on blindness prevalence based on subjective visual acuity measurement
- published in English

**Exclusion**
- undertaken in specific populations (e.g. in hospitals, ‘institutionalised’, ‘diabetics’)
- only included children
- only reported disease-specific blindness prevalence
- only reported blindness as an explanatory variable rather than as an outcome

### Data collection

Following discussion, we considered three of the 32 STROBE items not applicable to blindness prevalence reporting *a priori*. *Sensitivity analysis* (item 12e) was considered not applicable because we were focused on the reporting of the main result; *choice of category boundaries for continuous variables* (item 16b) because blindness (the outcome) is categorical and dichotomous (i.e. blind or not blind); and *translating relative risk into absolute risk* (item 16c) because the outcome was a prevalence rate, not a measure of association. The remaining 29 items were converted to questions to assist with consistent assessment. Three authors (JR, JP, VJ) independently reviewed one manuscript (not included in the sample) against the 29 STROBE items to develop the questions and corresponding reviewer guidelines through discussion and consensus. Five of the included manuscripts were then reviewed by the same three authors to clarify any discrepancies in application of the questions and finalise the guidelines. Each remaining manuscript in the sample was then reviewed by two authors (JR, JP, VJ, AP), with one author (JR) reviewing all. Discrepancies were resolved by discussion and consensus. In addition to the STROBE items, each included study was checked for a reference to STROBE, and whether the Rapid Assessment of Avoidable Blindness (RAAB) methodology was used. The 2015 SCImago Journal Rank (SJR) quartile was extracted for each ophthalmology journal with an included study (http://www.scimagojr.com) and categorised as ophthalmology journal top-quartile (Q1), ophthalmology journal outside top-quartile (Q2–4), or not ophthalmology journal.

Data were collated using an Excel spreadsheet (Microsoft Corp, Redmond, Washington). Each question was categorized as ‘yes’ (met criteria), ‘no’ (did not meet criteria) or ‘not applicable’. We did not include a ‘partly’ option, to avoid it being used to negate having to choose between yes and no.[11] Where a STROBE item contained several components, data were collected for each component. For example, item 5 relates to reporting “….setting, location and relevant dates…..” so data were collected separately on i) setting/location and ii) relevant dates. For item 5 to be assessed as adequate, both components needed to be reported.

To assess the extent to which STROBE was referred to by the journals in which included studies were published, two authors (JR and AP) checked the publicly available instructions for authors and reviewers. Each set of accessible instructions was categorised as STROBE being: required, recommended, listed (without recommendation), or not mentioned.

### Analysis

Analysis was completed using Excel and Stata 12.0 (StataCorp LP, TX). Descriptive analysis included the number and proportion of manuscripts reporting i) each of the 29 STROBE items, ii) use of RAAB methodology and iii) use of STROBE to guide reporting; the number and proportion of manuscripts published in the top-quartile of ophthalmology journals; and the number of journals including STROBE in instructions to i) authors and ii) reviewers.

The ‘Completeness of reporting (COR) score’ for each manuscript was calculated as the ‘yes’ answers as a proportion of the ‘yes + no’ answers: COR score (%) = [yes / (yes + no)] * 100. Consequently, the ‘not applicable’ items did not impact results.

Normality of COR scores was confirmed using distribution and probability plots. The mean COR score and 95% confidence interval (CI) were calculated for each category of author and reviewer instructions (i.e. required, recommended, listed, not mentioned, not accessible); use of RAAB methodology (used RAAB or other methodology); and publication in the top-quartile of ophthalmology journals (in the top-quartile, not in the top-quartile, not in an ophthalmology journal). One-way ANOVA or two-sample t-test was used to test for a difference between the mean scores of the categories. The median and inter-quartile range of the COR score within each manuscript section was calculated.

#### Sensitivity analysis

Some of the studies published in 2008 may have been prepared and reviewed prior to the dissemination of STROBE in 2007, so we performed sensitivity analysis to assess whether the inclusion of studies published in 2008 impacted our results. We calculated the COR score for studies published in 2008 and published after 2008, and used the two-sampled t-test to assess the statistical significance of the difference between mean COR scores in the two time periods.

## Results

### Summary of included studies

**Fig 1:**
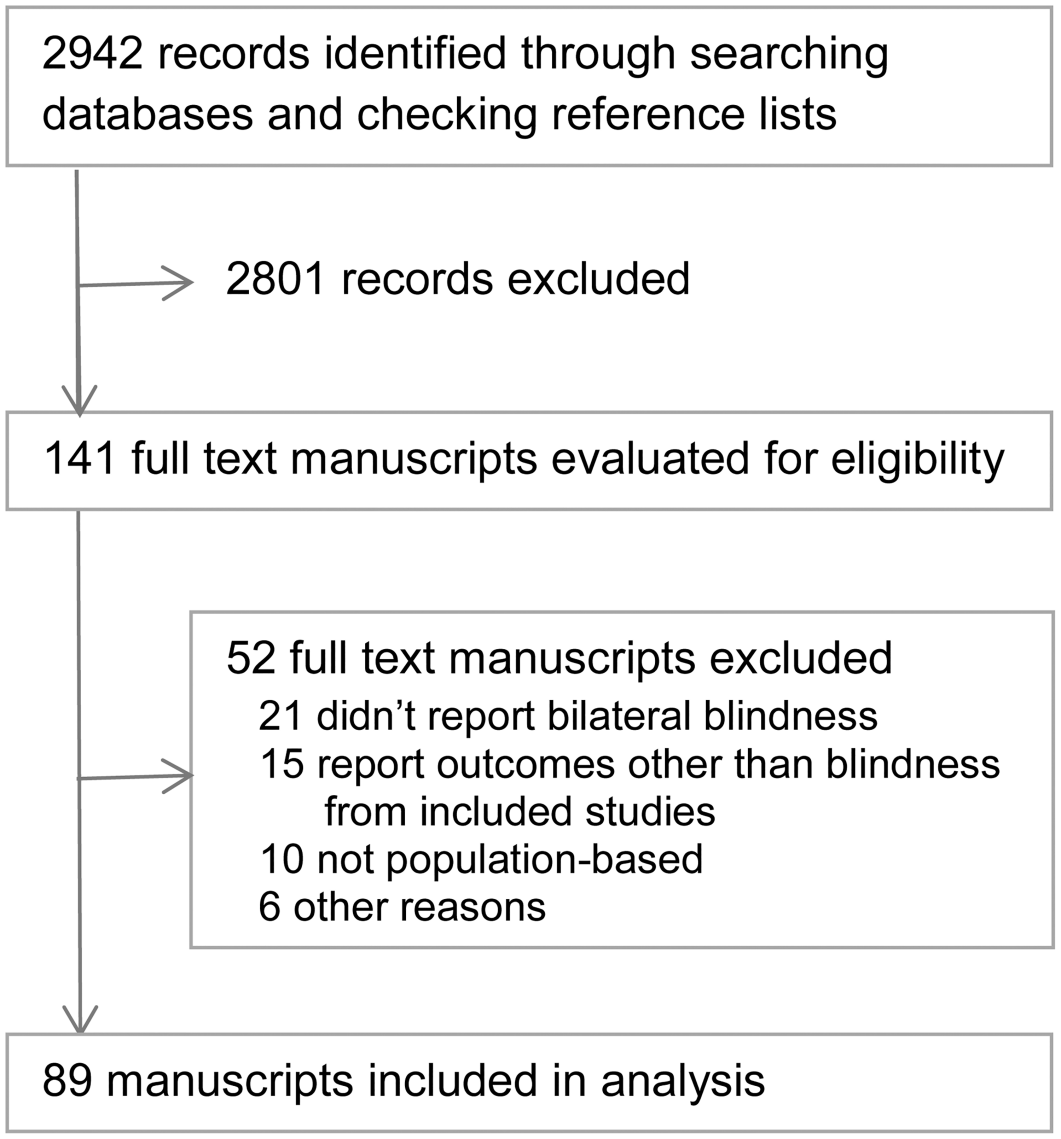
Summary of study search and selection.

The search identified 2942 studies (Fig 1), of which 89 met the inclusion criteria (listed in S1 Table). The studies were undertaken in 32 countries and published in 37 different journals. Eight of the 37 journals were in the top-quartile of ophthalmology journals (SJR quartile), and published almost half of the included studies (n = 42). More than one-third of studies (37%, n = 33) reported using the RAAB protocol. No manuscript reported using STROBE to guide reporting.

### Journals and STROBE

Of the 37 journals that published included studies, ten (27%) mentioned STROBE in instructions to authors—STROBE was listed without further instruction in four journals, recommended in five journals, and required by one journal (PLOS ONE). Reviewer instructions were available for 15 (41%) journals; 12 of these did not mention STROBE, two listed STROBE without further instruction and one (PLOS ONE) required adherence to reporting guidelines (including STROBE) for publication (Table 1). Only one of the eight top-ranked ophthalmology journals (that published three included studies) recommended the use of STROBE by authors, and none with reviewer comments available referred to STROBE. In contrast, of the 15 ophthalmology journals outside the top-quartile, four journals (publishing seven studies) listed STROBE and two journals (publishing four studies) recommended STROBE in instructions to authors; none mentioned STROBE in available reviewer instructions.

**Table 1:** Completeness of reporting (COR) score for journals publishing prevalence of blindness studies, by inclusion of STROBE in instructions for authors and reviewers, and ophthalmology SCImago Journal Rank.

	Number of journals	Number of manuscripts	Mean COR score* (95%CI), %
**Author instructions**			
STROBE required	1	10	68.2 (64.0–72.4)
STROBE recommended	5	8	53.1 (43.6–62.6)
STROBE listed	4	7	60.2 (52.3–68.0)
STROBE not mentioned	27	64	60.9 (57.4–64.3)
**Reviewer instructions**			
STROBE required	1	10	68.2 (64.0–72.4)
STROBE recommended	-	-	-
STROBE listed	2	2	45.0 (16.7–73.3)
STROBE not mentioned	12	26	64.6 (59.8–69.5)
Instructions not accessible	22	51	58.2 (54.5–62.0)
SCImago Journal Rank 2015**			
Ophthalmology top-quartile, Q1	8	42	66.1 (62.4–69.9)
Ophthalmology Q2–4	15	22	55.3 (51.0–59.7)
Not ophthalmology	14	25	57.2 (51.5–62.8)
**Total**	37	89	60.9 (58.1–63.7)

CI: confidence interval

*Completeness of reporting (COR) score is the proportion of the 29 included items adequately reported (items 12e, 16b and 16c removed *a priori* as they were considered not applicable to reporting blindness prevalence surveys).

** available at http://www.scimagojr.com

### Completeness of reporting

COR scores ranged from 30.8% to 88.9%, with a mean of 60.9% (95%CI 58.1–63.7%; Table 1). The mean COR score of studies that used RAAB methodology (58.8%, 95%CI 54.9–62.8%) was not different to studies using other methodologies (62.2%, 95%CI 58.4–65.9%; t(87) = 1.15, p = 0.25). The mean COR score of studies published in the top-quartile of ophthalmology journals was higher than studies published in lower-ranked ophthalmology journals or non-ophthalmology journals (Table 1; F(2,86) = 7.06, p = 0.001).

Inclusion of STROBE in instructions to authors did not tend to improve reporting—the mean score of studies published in journals with instruction to authors that required, recommended or listed STROBE (61.1%, 95%CI 56.4–65.8%) was not different to the score for studies published in journals not mentioning STROBE (60.9%, 95%CI 57.4–64.3%; t(87) = 0.09, p = 0.93); the mean score of studies published in the journal requiring STROBE (68.2%, 95%CI 64.0–72.4%) was higher than the score for the other studies combined (60.0%, 95%CI 57.0–63.1%) but this was not statistically significant (t(87) = 1.87, p = 0.065). Inclusion of STROBE in reviewer instructions also failed to improve reporting (64.3%, 95%CI 57.1–71.6% for required/recommended/listed versus 64.6%, 95%CI 59.8–69.5% for not mentioned; t(36) = 0.07, p = 0.95).

Sensitivity analysis showed there was no difference in the mean COR score of those studies published during 2008 and those published after 2008 (t(87) = 0.99, p = 0.32).

### Reporting of STROBE items

Nine items were reported in 90% or more of the included manuscripts (Table 2): providing an informative abstract (item 1b, 97%), scientific background (item 2, 96%) and study objectives (item 3, 99%); presenting key elements of study design (item 4, 91%); describing eligibility criteria (item 6, 92%); providing characteristics of study participants (item 14a, 90%) and number of outcome events (item 15, 91%); and summarising (item 18, 90%) and interpreting (item 20, 94%) results.

**Table 2:** Reporting of STROBE items in 89 blindness prevalence surveys published between January 2008 and March 31 2016.

STROBE Item	STROBE Description	Question answered	Individual component adequate			Overall item adequate* n (%)
			NA	No	Yes	
**Abstract**						
**1a**	Indicate the study’s design with a commonly used term in the title or the abstract	Is the design referred to as either ‘cross-sectional’ or ‘population-based survey’ in the title or abstract?	-	40 (45)	49 (55)	49 (55)
**1b**	Provide in the abstract an informative and balanced summary of what was done and what was found	Does the abstract provide an informative summary of what was done and found?	1 (1)	3 (3)	85 (96)	85 (97)
**Introduction**						
**2**	Explain the scientific background and rationale for the investigation being reported	Is the scientific background and rationale for the investigation reported?	-	4 (4)	85 (96)	85 (96)
**3**	State specific objectives, including any pre-specified hypotheses	Are objectives and /or hypotheses reported?	-	1 (1)	88 (99)	88 (99)
**Methods**						
**4**	Present key elements of study design early in the paper	Are the key elements of the study design presented before the end of methods?	-	8 (9)	81 (91)	81 (91)
**5**	Describe the setting, locations, and relevant dates, including periods of recruitment, exposure, follow-up, and data collection	Are the setting/locations reported?	-	6 (7)	83 (93)	71 (80)
		Are the dates of the survey reported?	-	13 (15)	76 (85)	
**6**	Give the eligibility criteria, and the sources and methods of selection of participants	Are eligibility criteria, and sources and methods of selection of participants provided?	-	7 (8)	82 (92)	82 (92)
**7**	Clearly define all outcomes, exposures, predictors, potential confounders, and effect modifiers. Give diagnostic criteria, if applicable	Is the outcome (i.e. blindness) defined in the introduction or methods, or the 1st time it is mentioned in results (e.g. <3/60 in the better eye)?	-	7 (8)	82 (92)	28 (31)
		Are diagnostic criteria for blindness described (i.e. how many letters on the line were correct/incorrect to be allocated to that level of vision)?	-	60 (67)	29 (33)	
**8**	For each variable of interest, give sources of data and details of methods of assessment (measurement). Describe comparability of assessment methods if there is more than one group	Is vision assessment described (in sufficient detail to be replicated)?	-	49 (55)	40 (45)	24 (27)
		If blindness is reported by age groups, is the method by which age was ascertained described?	32 (36)	38 (43)	19 (21)	
		If blindness is reported by another social variable (e.g. education), is the method by which this was ascertained described?	54 (61)	20 (22)	15 (17)	
**9**	Describe any efforts to address potential sources of bias	Was there any effort to address potential sources of:	-	23 (26)	66 (74)	33 (37)
		–selection bias when selecting clusters?				
		–selection bias when selecting individuals within clusters?	-	31 (35)	58 (65)	
		–measurement bias? (e.g. inter-rater agreement)	-	45 (51)	44 (49)	
**10**	Explain how the study size was arrived at	Was the method of establishing the study size explained AND the calculated size provided?	-	29 (33)	60 (67)	60 (67)
**11**	Explain how quantitative variables were handled in the analyses. If applicable, describe which groupings were chosen and why	Did they explain how quantitative variables were handled in the analysis?	-	46 (52)	43 (48)	43 (48)
**12a**	Describe all statistical methods, including those used to control for confounding	Were statistical methods for calculating blindness prevalence described?	-	48 (54)	41 (46)	41 (46)
**12b**	Describe any methods used to examine subgroups and interactions	If analysis is undertaken to compare blindness in subgroups, is the analysis explained in methods?	48 (54)	13 (15)	28 (31)	28 (68)
**12c**	Explain how missing data were addressed	(In the methods) is it reported how missing data were addressed?	-	86 (97)	3 (3)	3 (3)
**12d**	If applicable, describe analytical methods taking account of sampling strategy	If age standardization is reported in results, is the process explained in methods?	49 (55)	26 (29)	14 (16)	14 (34)
**Results**						
**13a**	Report numbers of individuals at each stage of study	Are the number of people enumerated and the number examined provided?	-	12 (13)	77 (87)	77 (87)
**13b**	Give reasons for non-participation at each stage	Are the reasons for non-participation provided?	-	50 (56)	39 (44)	39 (44)
**13c**	Consider use of a flow diagram†	Was a flow diagram used?	87 (98)	-	2 (2)	-
**14a**	Give characteristics of study participants (eg demographic, clinical, social)	Were participants described by (at least) age and sex?	-	9 (10)	80 (90)	80 (90)
**14b**	Indicate number of participants with missing data for each variable of interest	Are the number of participants with missing vision data reported (or can we tell from the data presented)?	-	60 (67)	29 (33)	29 (33)
**15**	Report numbers of outcome events or summary measures	Are the number of blind participants reported?	-	8 (9)	81 (91)	81 (91)
**16a**	Give unadjusted estimates and, if applicable, confounder-adjusted estimates and their precision (eg, 95% confidence interval).	Did they report [unadjusted and/or adjusted prevalence estimate] AND 95%CI?	1 (1)‡	14 (16)	74 (83)	74 (84)
**17**	Report other analyses done—eg analyses of subgroups and interactions, and sensitivity analyses	Was any analysis undertaken to compare blindness in subgroups?	1 (1)‡	48 (54)	40 (45)	40 (45)
**Discussion**						
**18**	Summarise key results with reference to study objectives	Are key results summarised with reference to the study objectives?	-	9 (10)	80 (90)	80 (90)
**19**	Discuss limitations of the study, taking into account sources of potential bias or imprecision. Discuss both direction and magnitude of any potential bias	Did they discuss the limitations of the study, taking into account sources of potential bias/ imprecision (both direction and magnitude) in relation to: –sample selection/representativeness;	-	70 (79)	19 (21)	15 (17)
		–other sources of potential bias or imprecision	-	48 (54)	41 (46)	
**20**	Give a cautious overall interpretation of results considering objectives, limitations, multiplicity of analyses, results from similar studies, and other relevant evidence	Did they provide an overall interpretation of results considering objectives, analyses, results from similar studies and other relevant evidence?	-	5 (6)	84 (94)	84 (94)
**21**	Discuss the generalisability (external validity) of the study results	Did they discuss the generalisability (external validity) of the study results?	-	62 (70)	27 (30)	27 (30)
**22**	Give the source of funding and the role of the funders for the present study	Is the funding source provided?	-	20 (22)	69 (78)	20 (22) §
		Is the role of the funder provided?	6 (7)	69 (78)	14 (17)	

Items 12e, 16b, 16c removed *a priori* as they were considered not applicable to reporting blindness prevalence surveys.

*to be assessed as a yes, individual components must be assessed as yes; NA not included; proportion (%) = yes/(yes+no).

^†^ as enumeration and participation were the only stages involved, we categorized this as yes or NA.

^‡^ one study found no blind participants (only vision impaired) so could not report this.

^§^ The role of funders was NA for 6 studies that received no specific funding; these studies were included in the numerator for the overall assessment of this item.

Seven items were reported in less than one-third of manuscripts: defining outcomes and diagnostic criteria (item 7, 31%); describing methods of measurement (item 8, 27%); explaining how missing data were managed (item 12c, 3%); reporting missing data (item 14b, 33%); discussing study limitations (item 19, 17%) and generalisability (item 21, 30%); and providing the name and role of the funder (item 22, 22%). Partial reporting within a number of these items reduced the overall score. For example, for item 7 blindness was commonly defined (in 92% of manuscripts) while diagnostic criteria were not (33%); and for item 22 the funder was often named (78%) but their role was not defined (17%; Table 2).

Overall, the extent of reporting varied notably across and within manuscript sections, with applicable items being most commonly reported in the Introduction (median COR Score = 97%; 2 items: 99% and 96%), followed by the Abstract (76%; 2 items: 97% and 55%), Results (65%, IQR 41–87), Methods (47%, IQR 33–71) and Discussion (51%, IQR 22–90).

## Discussion

The WHO’s global eye health action plan has called for more blindness prevalence surveys to be undertaken in LMICs.[2] Our results indicate that reporting of such surveys must be improved to maximise effective translation of the findings, and to aid synthesis. No single blindness prevalence manuscript published since the dissemination of STROBE adhered to more than 90% of STROBE items, and no single STROBE item was adequately addressed across all manuscripts (Table 2). Reporting was weakest in the Methods and Discussion sections. If authors improved adherence to STROBE, readers would better understand what was done and therefore whether the survey findings (i.e. blindness prevalence estimates) are valid.[12] Further, better adherence to STROBE would enable evidence-informed planning and monitoring of eye care services, and more accurate global estimates when results are synthesised.

Journal endorsement and use of reporting guidelines are expected to improve completeness of reporting. However, with the exception of CONSORT (CONsolidated Standards of Reporting Trials), few evaluations have assessed whether journal endorsement of guidelines improves reporting.[13] The CONSORT guidelines were launched in 1997 to improve reporting of randomised control trials (RCTs). A systematic review found that journals endorsing CONSORT improved reporting of RCTs, but the review also identified a gap between journals endorsing CONSORT and consistently implementing it.[14] Our results reflect a similar gap for STROBE—endorsement of STROBE in a journal’s instructions for authors and reviewers was not reflected by better reporting of the blindness prevalence surveys they published (Table 1). This lack of translation suggests that endorsement of reporting guidelines without enforcement by journal editors and reviewers is ineffectual. Some journals are trying to strengthen author application of STROBE. For example, The BMJ and PLOS Medicine require authors to complete the STROBE checklist to identify accomplishment of each item, with the checklist published alongside the study.[15, 16] This strategy could be implemented by ophthalmology journals, and further strengthened by requiring reviewers to assess adherence to STROBE when undertaking reviews.[17] We identified weaker reporting in lower-ranked ophthalmology journals (2015 SJR quartiles 2−4), suggesting these journals may benefit most from the use of STROBE to assist authors and reviewers.

STROBE development intentionally avoided being prescriptive,[4] which resulted in generalized guidelines,[12] some of which are not applicable to blindness prevalence surveys. We omitted three items from our assessment *a priori*, and others (such as the use of a flow diagram [13c]) seem less applicable after undertaking the review. The COR score calculation gave equal weight to each item in the STROBE checklist. As some items are arguably less important for reporting blindness prevalence surveys, the COR score is an imperfect summary measure. With the anticipated increase in surveys in the coming years, development of specific reporting guidelines for blindness prevalence surveys may be beneficial, and could include the identification of ‘essential’ items, as well as items that are not applicable. Fortunately, the process followed to develop such guidelines in other fields are available from the Equator Network (http://www.equator-network.org/). Until specific guidelines become available, the existing STROBE checklist can be used by authors of all blindness prevalence surveys.

Excluding some STROBE items from our assessment limits the comparability of our findings with other studies. Also, few studies report analysis for cross-sectional surveys separately to cohort and case-control studies. Despite these differences, our mean COR score (60.9%, 95%CI 58.1–63.7%) was similar to equivalent rates of ‘yes’/ ‘applicable’ items reported from STROBE assessment in diverse fields including orthopaedics (58%),[18] hand surgery (58% post-STROBE dissemination),[19] and dermatology (58% post-STROBE).[20]

RAAB surveys are the most commonly published blindness prevalence survey methodology. Reasons for this include that RAABs are shorter and less expensive than traditional surveys; the protocol and analysis software are readily available;[21] and RAABs have been designed to be implemented by local eye health workers following standardised training.[22] We found that the completeness of reporting of RAAB and non-RAAB surveys was similar. With the number of RAABs set to increase in the coming years, expanding the focus on reporting and the use of guidelines during RAAB training is a promising strategy to improve reporting of blindness prevalence surveys. In addition, ongoing monitoring of reporting quality may be beneficial, using the COR score (or similar), as well as graphical displays.

This analysis must be interpreted in the context of its limitations. Assessment of included studies was unblinded, which may have introduced bias through presumed reputation of some authors.[23] This potential for bias was lessened by two of the four reviewers being outside the field of eye care. Reviewer fatigue may also have been an issue, as data collection took place over a five-month period―we attempted to reduce this by developing assessment guidelines, using two reviewers for each study, and requiring any discrepancy to be discussed and resolved. In addition to English language studies, the search found 12 non-duplicate studies in Chinese and four in Spanish that we were unable to assess. The STROBE statement is available in these languages,[24] and could be applied by researchers fluent in reading them. Finally, we were unable to assess whether STROBE was included in instructions to authors at the time of submission of each individual manuscript. We believe this had little influence on our results―less than one-third of journals referred to STROBE in 2016, and it is unlikely that journals previously included STROBE in their instructions and subsequently removed it.

While not a limitation, to assist interpretation of our results it must be noted that we assessed completeness of reporting and not methodological quality or risk of bias of the included studies.[5] That is, we recorded whether an item was reported in sufficient detail to enable appraisal (e.g. steps taken to reduce bias in cluster selection), not whether the methods employed were sufficient to achieve the specific aim (e.g. avoid selection bias). Further, it is possible some of the checklist items were fulfilled during study design, implementation or analysis, but without complete reporting this remains unknown.

A scan of the literature suggests this is the first analysis of completeness of reporting of blindness prevalence surveys. It has revealed strong reporting in some areas, with other areas in need of improvement. The anticipated increase in surveys in LMICs in the coming years[2] provides the impetus for stakeholders to identify strategies to improve the quality of reporting and therefore increase the utility of survey findings. Supporters of research (international and national non-government organisations as well as academic institutions) could extend support beyond study implementation to also strengthen local research capabilities, including reporting. Endorsement of STROBE by journals should help, but only if its use is consistently enforced by editorial staff and reviewers. We recommend that ophthalmology journals adopt the STROBE checklist and ensure it is used by authors and reviewers.

## Supporting information

S1 File Medline search strategy. (PDF)

S2 File STROBE and blindness prevalence surveys data. (XLSX)

S1 Table List of blindness prevalence studies included in the evaluation of STROBE adherence. (PDF)
