# Supplementary material for: Using the STROBE statement to assess reporting in blindness prevalence surveys in low and middle income countries
Source: PLoS One. 2017 May 8;12(5):e0176178. doi: 10.1371/journal.pone.0176178 (PMC5421753; doi:10.1371/journal.pone.0176178)
Supplement: S1 File — (PDF) [file pone.0176178.s001.pdf]

Search History (4) View Saved

# ▲	Searches	Results	Type	Actions	Annotations
1	(blindness or vis* impairment or low vision).mp. [mp=title, abstract, original title, name of substance word, subject heading word, keyword heading word, protocol supplementary concept word, rare disease supplementary concept word, unique identifier]	43131	Advanced	Display Results | More	
2	(prevalence or rapid assessment or population-based).mp. [mp=title, abstract, original title, name of substance word, subject heading word, keyword heading word, protocol supplementary concept word, rare disease supplementary concept word, unique identifier]	625451	Advanced	Display Results | More	
3	1 and 2	4054	Advanced	Display Results | More	
4	limit 3 to yr="2008 -Current"	2007	Advanced	Display Results | More	
