# Supplementary material for: Using the STROBE statement to assess reporting in blindness prevalence surveys in low and middle income countries
Source: PLoS One. 2017 May 8;12(5):e0176178. doi: 10.1371/journal.pone.0176178 (PMC5421753; doi:10.1371/journal.pone.0176178)
Supplement: S1 Table — (PDF) [file pone.0176178.s003.pdf]

S1 Table: List of blindness prevalence studies included in the evaluation of STROBE adherence.

Citation
Al-Akily S, *et al.* Causes of blindness in people aged 50 years and over: community-based versus hospital-based study. Eastern Mediterranean Health Journal 2010;16(9):942-6.
Al-Khatib T, *et al.* Rapid assessment of avoidable blindness in Amran and Lahj governorates of Yemen. Sudanese Journal of Ophthalmology 2013;5(1):9.
Araujo Filho A, *et al.* Prevalence of visual impairment, blindness, ocular disorders and cataract surgery outcomes in low-income elderly from a metropolitan region of Sao Paulo--Brazil. Arquivos Brasileiros de Oftalmologia 2008;71(2):246-53.
Arieta C, *et al.* Cataract remains an important cause of blindness in Campinas, Brazil. Ophthalmic Epidemiology 2009;16(1):58-63.
Bejiga A, *et al.* Cataract surgical coverage and outcome in Goro District, Central Ethiopia. Ethiopian Medical Journal 2008;46(3):205-10.
Binnawi K, *et al.* Prevalence and causes of blindness and visual impairment in population aged 50 years and over in North Kordofan State, Sudan. Albasar Int J Ophthalmol 2015;3:6-10.
Binnawi K, *et al.* Prevalence and causes of blindness: Results from the rapid assessment of avoidable blindness survey in Gezira state, Sudan. Sudanese Journal of Ophthalmology 2013;5(1):17.
Budenz D, *et al.* Blindness and visual impairment in an urban West African population: the Tema Eye Survey. Ophthalmology 2012;119(9):1744-53.
Chiang F, *et al.* Rapid Assessment of Avoidable Blindness in the Occupied Palestinian Territories. Plos One 2010;5(7); e11854.
Cockburn N, *et al.* Prevalence, Causes and Socio-Economic Determinants of Vision Loss in Cape Town, South Africa. PLoS One 2012;7(2):e30718.
Dean W, *et al.* Follow-Up Survey of Cataract Surgical Coverage and Barriers to Cataract Surgery at Nkhoma, Malawi. Ophthalmic Epidemiology 2011;18(4):171-8.
Deepthi R, *et al.* Visual and hearing impairment among rural elderly of south India: A community-based study. Geriatrics and Gerontology International 2012;12(1):116-22.
Dhake PV, *et al.* Prevalence and causes of avoidable blindness and severe visual impairment in a tribal district of Maharashtra, India. Oman Journal of Ophthalmology 2011;4(3):129-34.
Duerksen R, *et al.* Review of blindness and visual impairment in Paraguay: changes between 1999 and 2011. Ophthalmic Epidemiology 2013;20(5):301-7.
Dulal S, *et al.* Prevalence of blindness and visual impairment and its causes among people aged 50 years and above in Karnali Zone, Nepal. Nepalese Journal of Ophthalmology 2012;4(2):282-7.
Edussuriya K, *et al.* The prevalence and causes of visual impairment in central Sri Lanka the Kandy Eye study. Ophthalmology 2009;116(1):52-6.
Feghhi M, *et al.* Prevalence and causes of blindness and low vision in Khuzestan province, Iran. Journal of Ophthalmic & Vision Research 2009;4(1):29-34.
Govekar KP, Sharma MK. Blindness and visual impairment in the Delhi region. National Journal of Community Medicine 2014;5(4):370-2.
Govendar P, *et al.* Rapid assessment of avoidable blindness in the northern eThekwini district of KwaZulu-Natal Province, South Africa. African Vision and Eye Health 2015;74(1)

Citation
Gupta N, *et al.* Rapid Assessment of Visual Impairment in urban population of Delhi, India. PLoS One 2015;10(4); e0124206.
Gurudasani B, *et al.* Prevalence of Cataract and Cataract Blindness in Wardha District. Scholars Journal of Applied Medical Sciences 2014;2(4C):1361-6.
Guruprasad B, *et al.* Changing scenario of cataract blindness in Kolar District, Karnataka, South India. The utility of rapid assessment of avoidable blindness in reviewing programs. Ophthalmic Epidemiology 2013;20(2):89-95.
Habiyakire C, *et al.* Rapid assessment of avoidable blindness and cataract surgical services in Kilimanjaro region, Tanzania. Ophthalmic Epidemiology 2010;17(2):90-4.
Harnagle R, *et al.* A prevalence study of visual impairment and blindness among elderly in an urban slum community of Pune cantonment, India. Research Journal of Pharmaceutical, Biological and Chemical Sciences 2013;4(4):1452-61.
Hashemi H, *et al.* Visual impairment in the 40-to 64-year-old population of Shahroud, Iran. Eye 2012;26(8):1071-7.
Hashemi H, *et al.* The Prevalence and Causes of Visual Impairment and Blindness in a Rural Population in the North of Iran. Iran J Public Health 2015;44(6):855-64.
Huang S, *et al.* Prevalence and causes of visual impairment in Chinese adults in urban southern China. Archives of Ophthalmology 2009;127(10):1362-7.
Isipradit S, *et al.* The First Rapid Assessment of Avoidable Blindness (RAAB) in Thailand. PloS One 2014;9(12):e114245.
Jimenez-Corona A, *et al.* Social Determinants and Their Impact on Visual Impairment in Southern Mexico. Ophthalmic Epidemiology 2015;22(5):342-8.
Kalua K, *et al.* Findings from a rapid assessment of avoidable blindness (RAAB) in Southern Malawi. PLoS One 2011;6(4):e19226.
Kandeke L, *et al.* Rapid assessment of avoidable blindness in two northern provinces of Burundi without eye services. Ophthalmic Epidemiology 2012;19(4):211-5.
Katibeh M, *et al.* Prevalence and Causes of Visual Impairment and Blindness in Central Iran; The Yazd Eye Study. J Ophthalmic Vis Res. 2015;10(3):279–85.
Khanna RC. Prevalence and causes of blindness and visual impairment and their associated risk factors, in three tribal areas of Andhra Pradesh, India. PloS One 2014;9(7):e100644.
Kolawole O, *et al.* Cataract blindness in Osun state, Nigeria: results of a survey. Middle East African Journal of Ophthalmology 2012;19(4):364-71.
Kolawole O, *et al.* Survey of blindness and low vision in Egbedore, South-Western Nigeria. West African Journal of Medicine 2010;29(5):327-31.
Kyari F, *et al.* Prevalence of blindness and visual impairment in Nigeria: the National Blindness and Visual Impairment Study. Investigative Ophthalmology & Visual Science 2009;50(5):2033-9.
Lepcha N, *et al.* Rapid Assessment of Avoidable Blindness in Bhutan. Ophthalmic Epidemiology 2013;20(4):212-9.
Li E, *et al.* Prevalence of blindness and outcomes of cataract surgery in Hainan Province in South China. Ophthalmology 2013;120(11):2176-83.

Citation
Li J, *et al.* The prevalence and causes of visual impairment in an elderly Chinese Bai ethnic rural population: the Yunnan minority eye study. Investigative Ophthalmology & Visual Science 2012;53(8):4498-504.
Li L, *et al.* Prevalence and causes of visual impairment among the elderly in Nantong, China. Eye 2008;22(8):1069-75.
Li X, *et al.* Prevalence of blindness and low vision in a rural population in northern China: preliminary results from a population-based survey. Ophthalmic Epidemiology 2012;19(5):272-7.
Li Z, *et al.* Prevalence and causes of blindness and visual impairment among the elderly in rural southern Harbin, China. Ophthalmic Epidemiology 2008;15(5):334-8.
Li T, *et al.* Prevalence and Causes of Visual Impairment and Blindness in Shanxi Province, China. Ophthalmic Epidemiology 2015;22(4):239-45.
Liang Y, *et al.* Prevalence and Causes of Low Vision and Blindness in a Rural Chinese Adult Population The Handan Eye Study. Ophthalmology 2008;115(11):1965-72.
Lindfield R, *et al.* A rapid assessment of avoidable blindness in Southern Zambia. PLoS One 2012;7(6):e38483.
Marmamula S, *et al.* Rapid assessment of visual impairment (RAVI) in marine fishing communities in South India--study protocol and main findings. BMC Ophthalmology 2011;11:26.
Marmamula S, *et al.* Visual Impairment among Weaving Communities in Prakasam District in South India. PLoS One 2013;8(2);e55924.
Marmamula S, *et al.* Visual Impairment in the South Indian State of Andhra Pradesh: Andhra Pradesh - Rapid Assessment of Visual Impairment (AP-RAVI) Project. PLoS One 2013;8(7);e70120.
Mashayo E, *et al.* Prevalence of refractive error, presbyopia and spectacle coverage in Kahama District, Tanzania: a rapid assessment of refractive error. Clinical & Experimental Optometry 2015;98(1):58-64.
Mathenge W, *et al.* The Nakuru Posterior Segment Eye Disease Study Methods and Prevalence of Blindness and Visual Impairment in Nakuru, Kenya. Ophthalmology 2012;119(10):2033-9.
Minderhoud J, *et al.* Blindness and Visual Impairment in the Republic of Suriname. Ophthalmology 2015;122(10):2147-9.
Mörchen M, *et al.* Prevalence of Blindness and Cataract Surgical Outcomes in Takeo Province, Cambodia. The Asia-Pacific Journal of Ophthalmology 2015;4(1):25-31.
Mousa A, *et al.* Prevalence of Visual Impairment and Blindness in Upper Egypt: A Gender-based Perspective. Ophthalmic Epidemiology 2014;21(3):190-6.
Mpyet C, *et al.* Prevalence and Causes of Blindness and Visual Impairment in Plateau State, Nigeria. TAF Preventive Medicine Bulletin 2010;9(5):401-6.
Muhammad N, *et al.* Prevalence and causes of blindness and visual impairment in Sokoto State, Nigeria: Baseline data for Vision 2020: The right to sight eye care programme. Middle East African Journal of Ophthalmology 2011;18(2):123-8.
Muller A, *et al.* Results of a rapid assessment of avoidable blindness (RAAB) in Eritrea. Ophthalmic Epidemiology 2011;18(3):103-8.
Murthy GV, *et al.* Prevelence and causes of visual impairment and blindness in older adults in an area of India with a high cataract surgical rate. Ophthalmic Epidemiology 2010;17(4):185-95.

**Citation**
Marmamula S, *et al.* Rapid assessment of visual impairment (RAVI) in marine fishing communities in South India--study protocol and main findings. BMC Ophthalmology 2011;11:26.
Nangia V, *et al.* Visual impairment and blindness in rural central India: the Central India Eye and Medical Study. Acta Opthalmologica 2013;91(5):483-6.
Neena J, *et al.* Rapid Assessment of Avoidable Blindness India Study, Group. Rapid Assessment of Avoidable Blindness in India. PLoS One 2008;3(8):e2867.
Patil S, *et al.* Prevalence, causes of blindness, visual impairment and cataract surgical services in Sindhudurg district on the western coastal strip of India. Indian Journal of Ophthalmology 2014;62(2):240-5.
Polack S, *et al.* Rapid Assessment of Avoidable Blindness and Diabetic Retinopathy in Chiapas, Mexico. Ophthalmology 2012;119(5):1033-40.
Rabiu M, *et al.* Prevalence and Causes of Visual Impairment and Blindness, Cataract Surgical Coverage and Outcomes of Cataract Surgery in Libya. Ophthalmic Epidemiology 2013;20(1):26-32.
Rabiu M, *et al.* Prevalence of blindness and diabetic retinopathy in northern Jordan. European Journal of Ophthalmology 2015;25(4):320-7.
Rabiu M, *et al.* Rapid Assessment of Cataract Surgical Services in Birnin-Kebbi Local Government Area of Kebbi State, Nigeria. Ophthalmic Epidemiology 2008;15(6):359-65.
Rabiu M. Prevalence of blindness and low vision in north central, Nigeria. West African Journal of Medicine 2008;27(4):238-44.
Rajavi Z, *et al.* Rapid Assessment of Avoidable Blindness in Iran. Ophthalmology 2011;118(9):1812-8.
Ramke J, *et al.* Prevalence and causes of blindness and low vision among adults in Fiji. Clinical and Experimental Ophthalmology 2012;40(5):490-6.
Ramke J, *et al.* Prevalence and Causes of Blindness and Low Vision Revisited after 5 years of Eye Care in Timor-Leste. Ophthalmic Epidemiology 2012;19(2):52-7.
Randrianaivo J, *et al.* Blindness and cataract surgical services in Atsinanana region, Madagascar. Middle East African Journal of Ophthalmology 2014;21(2):153-7.
Rius A, *et al.* Prevalence of visual impairment in El Salvador: inequalities in educational level and occupational status. Pan American Journal of Public Health 2014;36(5):290-9.
Salomao S, *et al.* Prevalence and causes of vision impairment and blindness in older adults in Brazil: the Sao Paulo Eye Study. Ophthalmic Epidemiology 2008;15(3):167-75.
Sapkota YD, *et al.* The Prevalence of Blindness and Cataract Surgery in Rautahat District, Nepal. Ophthalmic Epidemiology 2010;17(2):82-9.
Sherchan A, *et al.* Blindness prevalence and cataract surgical coverage in Lumbini Zone and Chetwan District of Nepal. British Journal of Ophthalmology 2010;94(2):161-6.
Singh N, *et al.* Prevalence and causes of blindness and visual impairment and their associated risk factors, in three tribal areas of Andhra Pradesh, India. PLoS One 2014;9(7):e100644.
Song W, *et al.* Prevalence and causes of visual impairment in a rural North-east China adult population: a population-based survey in Bin County, Harbin. Acta Opthalmologica 2010;88(6):669-74.
Soori H, *et al.* Prevalence and causes of low vision and blindness in Tehran Province, Iran. Journal of the Pakistan Medical Association 2011;61(6):544-9.

Citation
Tang Y, *et al.* Prevalence and Causes of Visual Impairment in a Chinese Adult Population: The Taizhou Eye Study. Ophthalmology 2015;122(7):1480-8.
Thapa S, *et al.* Prevalence of visual impairment, cataract surgery and awareness of cataract and glaucoma in Bhaktapur district of Nepal: The Bhaktapur Glaucoma Study. BMC Ophthalmology 2011;11:2.
Wei M, *et al.* Prevalence and causes of visual impairment and blindness in Sichuan province of China. International Journal of Ophthalmology 2010;3(1):83-8.
Woldeyes A, Adamu Y. Gender differences in adult blindness and low vision, Central Ethiopia. Ethiopian Medical Journal 2008;46(3):211-8.
Wu M, *et al.* Rapid assessment of avoidable blindness in Kunming, China. Ophthalmology 2008;115(6):969-74.
Xiao B, *et al.* Rapid assessment of avoidable blindness in three counties, Jiangxi Province, China. British Journal of Ophthalmology 2010;94(11):1437-42.
Xiao B, *et al.* The Prevalence of Blindness, Visual Impairment and Cataract Surgery in Tuoketuo and Shangdu Counties, Inner Mongolia, China. Open Journal of Ophthalmology 2015;5(01):23-30.
Yaacov-Pena F, *et al.* Prevalence and causes of blindness in an urban area of Paraguay. Arquivos Brasileiros de Oftalmologia 2012;75(5):341-3.
Yao Y, *et al.* Prevalence of blindness and causes of visual impairment among adults aged 50 years or above in southern Jiangsu province of China. Pakistan Journal of Medical Sciences 2013;29(5);1203-7.
Zatic TB, *et al.* Rapid assessment of avoidable blindness and diabetic retinopathy in Republic of Moldova. British Journal of Ophthalmology 2015;99(6):832-6.
Zhang Y, *et al.* Prevalence of blindness and low vision: a study in the rural Heilongjiang Province of China. Clinical and Experimental Ophthalmology 2012;40(5):484-9.
Zhao J, *et al.* Prevalence of Vision Impairment in Older Adults in Rural China The China Nine-Province Survey. Ophthalmology 2010;117(3):409-U27.
Zhu M, *et al.* Visual impairment and spectacle coverage rate in Baoshan district, China: population-based study. BMC Public Health 2013;13:311.
